# Protocol to isolate dorsal root ganglion neurons from embryonic rats by immunopanning and characterize them using RNAscope and immunofluorescence

**DOI:** 10.1016/j.xpro.2025.103836

**Published:** 2025-05-21

**Authors:** Samuel Faraguna, Karina A. Peña, Husniye Kantarci

**Affiliations:** 1 Department of Neuroscience, University of Texas at Austin, Austin, TX, USA

**Keywords:** cell culture, cell isolation, developmental biology, *in situ* hybridization, neuroscience

## Abstract

Dorsal root ganglion (DRG) neurons innervate the periphery of the body to transmit somatosensory information, including touch, pain, and temperature. Here, we present a protocol for isolating rat DRG neurons using an immunopanning technique. We describe steps for dissecting DRGs from embryonic day (E)15 rat embryos, triturating ganglionic cells, and purifying DRG neurons. We also detail procedures for mRNA or protein quantification using RNAscope and immunofluorescence. This protocol has potential applications for studying DRG neurons during development and disease.

For complete details on the use and execution of this protocol, please refer to Kantarci et al.^1^

## Highlights

• Utilizing immunopanning to isolate dorsal root ganglion neurons from rat embryos
• Study of sensory neurons in cultures largely devoid of glia or other cell types
• Detection of proteins of interest via immunofluorescence in isolated neurons
• Guidance for using RNAscope to quantify target mRNA molecules expressed in neurons

## Abstract (editor highlights)

Publisher’s note: Undertaking any experimental protocol requires adherence to local institutional guidelines for laboratory safety and ethics.

## Graphical abstract

**Figure:**
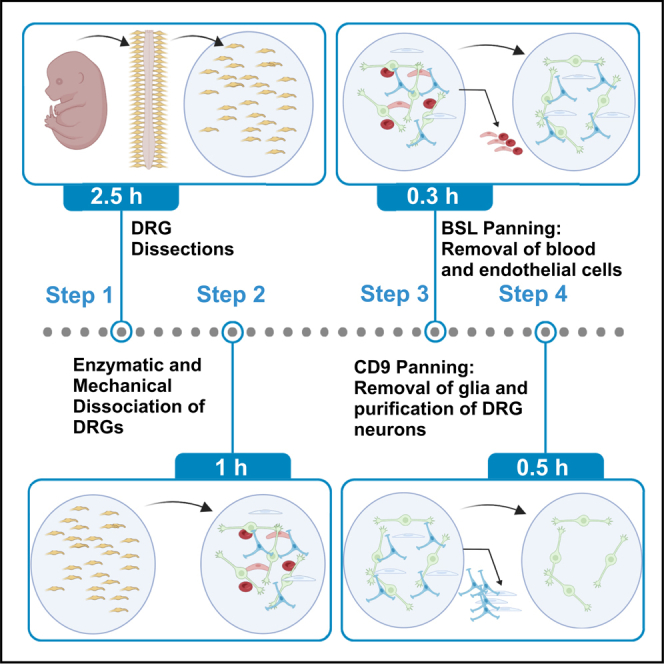

## Before you begin

The protocol below describes the steps of isolating DRG neurons from E15 rat embryos.^1^^,^^2^^,^^3^ The following reagents and materials need to be prepared prior to the beginning of the protocol. We suggest completing steps one day prior to the start of the procedure to ensure the success of the protocol. However, it is also feasible to do the BSL-1 or secondary antibody incubation of panning plates and laminin incubation of coverslips on the day of the immunopanning, for 2 h in a 37 °C incubator. Alternatively, the coating of panning plates with secondary antibodies can be done up to one week before the immunopanning experiment. Please refer to the key resources table for a list of reagents, and to the materials and equipment section for tables containing the recipes for preparing stock solutions for each reagent.

1. Prepare BDNF master and working stock solutions (See materials and equipment setup for recipe).
  a. Master Stock Protocol.
    i. (All on ice) Make 0.2% BSA by adding 1 mL of 4% BSA to 19 mL DPBS.
    ii. Filter through solution and put 250 μL of 0.2% of BSA on ice.
    iii. Spin down the 250 μg tube of BDNF for 1 min in a tabletop centrifuge at maximum speed at 18°C–25°C then resuspend gently with 250 μL of 0.2% BSA.
    iv. Make two 125 μL Master Stock aliquots in Low Protein Binding Tubes.
    v. Flash-freeze in liquid N_2_ and store in −80°C.
  b. Working Stock Protocol.
    i. On ice, chill 3 mL of 0.2% BSA and thaw a 125 μL Master Stock aliquot.
    ii. Add 2.375 mL of 0.2% BSA into Bijoux tube.
    iii. Add 125 μL of BDNF Master Stock and mix gently.
    iv. Aliquot 80 μL of BDNF solution per tube.
  c. Flash-freeze in liquid N_2_ and store in −80°C. Manufacturer guidelines suggest storing BDNF solutions at −80°C for up to 12 months for PeproTech products and up to 3 months for STEMCELL products.
2. Prepare NGF master and working stock solutions (See materials and equipment setup for recipe).
  a. Prepare NGF master stocks.
    i. Chill sterile filtered ddH_2_O on ice.
    ii. Spin down the 1 mg tube in a tabletop centrifuge at maximum speed and resuspend in 1 mL of the ddH_2_O via gentle trituration.
    iii. Make 5 tubes of 200 μL.
    iv. Flash-freeze in liquid N_2_.
  b. Prepare NGF working stocks.
    i. On ice, chill 2 mL of 0.2% BSA and thaw a 200 μL Master Stock aliquot.
    ii. Add 1800 μL of 0.2% BSA into a Bijoux tube.
    iii. Add 200 μL of NGF Master Stock and mix gently.
    iv. Aliquot 20 μL of NGF solution per tube.
    v. Flash-freeze in liquid N_2_. Manufacturer guidelines suggest storing NGF solutions at −80°C for up to 3 months for Bio-Rad and STEMCELL products.
3. Prepare NT-3 master and working stock solutions (See materials and equipment setup for recipe).
  a. Prepare NT-3 master stocks.
    i. (All on ice) Make 0.2% BSA by adding 1 mL of 4% BSA to 19 mL DPBS.
    ii. Filter through solution and put 500 μL of 0.2% of BSA on ice.
    iii. Spin down the 50 μg NT-3 tube in a tabletop centrifuge at maximum speed and resuspend in 50 μL of filtered and chilled ddH_2_O.
    iv. Dilute the solution by adding 450 μL of 0.2% BSA.
    v. Make ten 50 μL Master Stock aliquots in Low Protein Binding Tubes.
    vi Flash-freeze in liquid N_2_ and store in −80°C.
  b. Prepare NT-3 working stocks.
    i. On ice, chill 3 mL of 0.2% BSA and thaw a 50 μL Master Stock aliquot.
    ii. Add 4950 μL of 0.2% BSA into Bijoux tube.
    iii. Add 50 μL of NT-3 Master Stock and mix gently.
    iv. Aliquot 80 μL of NT-3 solution per tube.
    v. Flash-freeze in liquid N_2_ and store in −80°C. Manufacturer guidelines suggest storing NT-3 solutions at −80°C for up to 3 months for PeproTech and STEMCELL products.
4. Prepare Sato Solution.
  a. Prepare Progesterone stock by dissolving 2.5 mg of progesterone into 100 μL of EtOH.
  b. Prepare sodium selenite stock by dissolving 4 mg of Sodium Selenite and 10 μL of 1 N NaOH into 10 mL Neurobasal media.
    ***Note:*** Do not reuse progesterone and sodium selenite stocks; make fresh each time.
  c. Dissolve 1.0 g of transferrin, 160 mg of putrescine, and 1.0 g of BSA into 100 mL of Neurobasal medium.
  d. Add 25 μL of fresh progesterone stock and 1000 μL of fresh sodium selenite stock into the solution.
  e. Filter through pre-rinsed 0.22 μm filter and make 200 μL aliquots.
  f. Store at −20°C.
5. Begin preparing the panning plates (first part out of two).
  a. Prepare BSL-1 panning plate for depletion of blood and endothelial cells.
    i. Coat 15 cm Petri dish (VWR, 25373-187) with 60 μL of 2 mg/mL BSL-1 (Vector Labs, L-1100) diluted in 20 mL Phosphate buffered saline (PBS) (Sigma-Aldrich, P3813).
      ***Note:*** Tilt plate and pipette directly onto edge of plate, making sure to pipette up and down in solution 2–3 times to mix, set the plate down immediately and shake to cover the surface with the antibody solution.
      **CRITICAL:** Both plates must be shaken front-back and side-to-side once placed on a new flat surface so that solution evenly coats entirety of the dish.
  b. Prepare CD9 panning plate for depletion of glial cells.
    i. Coat 15 cm Petri dish with 90 μL of Goat-anti-mouse IgG+ IgM (H+L) secondary antibody (Jackson ImmunoResearch, 115-005-044) diluted in 20 mL of Tris-HCl (pH 9.5).
      ***Note:*** Tilt plate and pipette directly onto edge of plate, making sure to pipette up and down in solution 2-3 times to mix, set the plate down immediately and shake to cover the surface with the antibody solution.
      **CRITICAL:** Both plates must be shaken front-back and side-to-side once placed on a new flat surface so that solution evenly coats entirety of the dish.
  c. Incubate both plates at 4 °C for ≥16 h.
    ***Note:*** This is the first part out of two, the second half of preparation of immunopanning plates should be done on the day of the prep.
6. Prepare coverslips.
  a. Wash coverslips continuously in 100% ethanol (Carolina Bio, 633029) on a digital table shaker for 1 month at 150 rpm (ethanol was changed out once a day, but it is acceptable to skip a few changes).^4^^,^^5^
    ***Note:*** Alternatively, our research group has also successfully used Neuvitro coverslips (Neuvitro Cat# GG121.5PDL), or Ibidi USA dishes (Cat # 80607, 80807 or 81506) without washing in ethanol, provided the poly-D-lysine borate (PDL-borate, Sigma-Aldrich, P6407) and laminin coating steps described below are followed.
  b. Perform the coverslip preparation steps described below in a cell culture hood.
  c. Count out number of glass coverslips planning to use and rinse them with sterile water 3 times in a 15 cm Petri dish.
  d. After last rinse, aspirate off any remaining water and separate coverslips so they do not overlap each other.
  e. Allow coverslips to air dry completely (about 10 min) in a cell culture hood.
  f. Carefully add 100 μL 1× PDL-borate (PDL, Sigma-Aldrich, P6407) to the center of each coverslip; the PDL-borate solution should remain as a domed drop on the coverslip.
  g. Close the lid of the 15 cm Petri dish to prevent evaporation.
  h. Incubate for 30 min at 18°C–25°C.
  i. Remove PDL-borate and rinse coverslips 3 times with sterile water.
  j. Aspirate any remaining water from wells, being careful to keep coverslips centered and not touching the sides of the plate.
  k. Allow coverslips to air dry completely.
  l. Add 100 μL laminin solution (dilute 10 μL laminin in 5 mL neurobasal media) (Fisher Scientific, 340001002) to each coverslip.
    ***Note:*** Laminin solution should remain as a domed drop on the coverslips. If the surface tension breaks, carefully aspirate the laminin solution on the coverslip and try again.
  m. Gently place lid on well plate and let sit in 37°C incubator 16 to 24 h without disturbing, which could cause the laminin drops to get displaced off the coverslips.
    ***Note:*** Transfer coverslips to 24-well plates prior to plating cells following day.
7. Prepare DRG base media (80 mL).
  a. Add the following into sterile 150 mL MilliporeSigma Stericup (Fisher Scientific, S2GPU01RE) with 0.45 μm syringe filter for 80 mL media.
8. If making only 20 mL media, a Stericup, a 0.22 μm syringe filter (Fisher Scientific, SLHVR33RS) could also be used.
  a. 36.76 mL Dulbecco’s Modified Eagle’s Medium (DMEM) (Thermo Fisher, A1896701)
  b. 36.76 mL Neurobasal Medium (Thermo Fisher, 21103049)
  c. 800 μL insulin stock (Sigma-Aldrich, I2643)
  d. 800 μL 100× Sodium Pyruvate (Thermo Fisher, 11360070)
  e. 800 μL 100× Penicillin / Streptomycin (Thermo Fisher, 15140122)
  f. 800 μL SATO stock.
  g. 1600 μL NS-21 (R&D Systems, AR008).
  h. 800 μL 100× T3 stock (Sigma-Aldrich, T6397).
  i. 800 μL 100× L-glutamine (Thermo Fisher, 25030081).
  j. 80 μL N-acetyl-L-cysteine stock solution (Sigma-Aldrich, A8199).
9. Close top lid and use biosafety cabinet vacuum line to force solution through filter.
10. Remove and discard filter head then label bottle and screw lid on.
11. This media can be scaled up or down based on the number of well plates and frequency of feeding DRG neurons. Use 500 μL of media per well in a 24–well plate.
  a. Store at 4°C for up to 1 week.

### Institutional permissions

Use of animals follows the Administrative Panel guidelines on Laboratory Animal Care (IACUC) at UT Austin, in compliance with the United States department of Agriculture (USDA) and the National Institutes of Health (NIH) Office of Laboratory Animal Welfare and University policies and procedures. Any experiments that utilize animals require prior approval from the appropriate ethics committee at your research institution.

### Preparation on the day of the DRG immunopanning

**Timing: 1 h**

12. Prepare 20 mL of 0.2% Bovine Serum Albumin (BSA) solution.
13. Combine 1 mL 4% BSA solution (Sigma-Aldrich, A4161) and 19 mL DPBS. (Dulbecco’s Phosphate Buffered Saline Modified with Calcium, & Magnesium) (Fisher Scientific, SH30264FS).
14. Store in 4°C.
15. Prepare dissection plates.
  a. Add 15 mL of Leibovitz’s L-15 medium (Thermo Fisher, 11415064) to 15 cm Petri dish.
  b. Add 10 mL of Leibovitz’s L-15 medium to 10 cm Petri dish (VWR, 25382-166).
  c. Add 5 mL of PBS (modified with calcium & magnesium) to 6 cm Petri dish (VWR, 25373-085).
16. Coat panning plates with antibodies (second part out of two).
  a. Rinse BSL-1 plate with autoclaved PBS 3 times. Add 9 mL 0.2% BSA solution to BSL-1 plate.
  b. Rinse CD9 plate with autoclaved PBS 3 times. Add 10 mL 0.2% BSA solution + 60 μL anti-rat CD9 antibody (Fisher Scientific, BDB551808) to CD9 plate.
    **CRITICAL:** Both plates must be shaken front-back and side-to-side so that solution evenly coats entirety of dish.
  c. Incubate panning plates ≥2 h at 18°C–25°C.
17. In a 15 mL Falcon tube (Fisher Scientific, 352196), combine 10 mL DPBS + 6 μL 1 N NaOH (Millipore Sigma, S2770). Place tube in water bath set to 37°C to warm until ready to add Papain (Dissociation, step 7).

***Note:*** Use DPBS without calcium & magnesium (Thermo Fisher, 14190250) for papain steps only and modified DPBS with calcium & magnesium for all other steps.

***Note:*** In later steps during Dissociation step 16, the addition of L-cysteine to the papain solution activates the papain and causes the pH of the solution to become acidic, as indicated by the phenol-red-containing PBS solution turning yellow. NaOH is added preemptively to adjust the pH to neutral level.

## Key resources table

**Table:** 

REAGENT or RESOURCE	SOURCE	IDENTIFIER
**Antibodies**		

Goat-anti-mouse IgG + IgM (H+L) secondary antibody	Jackson ImmunoResearch, 1: 222	RRID:AB_2338451; Cat# 115-005-044
Anti-rat CD9 antibody	Fisher Scientific, 1: 166	RRID:AB_394262; Cat# 551808
Neurofilament	Sigma-Aldrich, 1:1,000	AB_477272; Cat# N4142
Anti Na_v_1.1	Alomone Labs, 1:1,000	RRID:AB_2040003; Cat# Asc-001
Anti Na_v_1.2	Alomone Labs, 1:1,000	RRID:AB_2040005; Cat# Asc-002
Anti Na_v_1.6	Alomone Labs, 1:1,000	RRID:AB_2040202; Cat# Asc-009
Anti Na_v_1.7	Alomone Labs, 1:1,000	RRID:AB_2040198; Cat# Asc-008
Anti Na_v_1.8	Alomone Labs, 1:1,000	RRID:AB_2040188; Cat# ASC-016
Anti Na_v_ 1.5	Alomone Labs, 1:1,000	RRID:AB_2040001; Cat# ASC-005
Anti Na_v_ 1.9	Alomone Labs, 1:1,000	RRID: AB_2340966; Cat #ASC-017-GP (formerly AGP-030)

**Chemicals, peptides, and recombinant proteins**		

BSL-1	Vector Labs	L-1100
PBS	Sigma-Aldrich	P3813
Modified DPBS	Fisher Scientific	14040133
Tris base	Fisher Scientific	BP1525
EtOH	Fisher Bioreagents	BP28184
PDL hydrobromide	Sigma-Aldrich	P6407
Laminin	R&D Systems	340001002
DMEM	Thermo Fisher Scientific	A1896701
Neurobasal medium	Thermo Fisher Scientific	21103049
Insulin	Sigma-Aldrich	CAS #11061680; I2643
Sodium pyruvate	Thermo Fisher Scientific	11360070
Penicillin/streptomycin	Thermo Fisher Scientific	15140122
Progesterone	Sigma-Aldrich	CAS #57830;P8783
Sodium selenite	Sigma-Aldrich	CAS #10102188; S5261
Putrescine dihydrochloride	Sigma-Aldrich	CAS #333937; P5780
NS-21/B27	R&D Systems	AR008
T3	Sigma-Aldrich	CAS #55061; T6397
L-glutamine	Thermo Fisher Scientific	25030081
N-acetyl-L-cysteine	Sigma-Aldrich	CAS #616911; A8199
BSA	Sigma-Aldrich	CAS #9048468; A4161
Leibovitz’s L-15 medium	Thermo Fisher Scientific	11415064
Phenol red	Sigma-Aldrich	P0290
NaOH	Thomas Scientific	C987H93
Papain	Fisher Scientific	CAS #9001734; NC9597281
EBSS	Sigma-Aldrich	E6267
MgSO4	Sigma-Aldrich	M1880
EGTA	Sigma-Aldrich	E4378
NaHCO3	Sigma-Aldrich	S8875
L-cysteine hydrochloride	Sigma-Aldrich	C7477
DNase	Worthington Biochemical	CAS #9003989; LS002007
Trypsin inhibitor	Worthington Biochemical	CAS #9035811; LS003086
Trypan blue	Thermo Fisher Scientific	15250061
BDNF	PeproTech	Cat# 450-02; Accession #:P23560
NT3	PeproTech	Cat# 450-03; Accession #:P20783
NGF	Bio-Rad	Cat# PMP04Z
FUDR	Sigma-Aldrich	CAS #50919; F0503-250MG
dmPGE_2_	Cayman	CAS #39746-25-3; Cat# 14750

**Critical commercial assays**		

RNAscope multiplex fluorescent reagent kit v.2	ACDBio	Cat# 323100

**Deposited data**		

Raw image files for Figure 2	Mendeley Data	https://data.mendeley.com/datasets/rxvfxkr6v9/1

**Experimental models: Organisms/strains**		

Sprague-Dawley timed-pregnant female rats with E15 embryos	Charles River Laboratories	001

**Software and algorithms**		

GraphPad Prism version 10.2.0	GraphPad Software, LLC	RRID:SCR_002798; http://www.graphpad.com
Fiji	Schindelin et al.^6^	RRID:SCR_002285; http://fiji.sc
MATLAB version: R2024b	The MathWorks, Inc.	RRID:SCR_001622; http://www.mathworks.com/products/matlab/
FishQuant	Mueller et al.^7^	http://code.google.com/p/fish-quant/
Cellpose	Stringer et al.^8^	RRID:SCR_021716; http://www.cellpose.org
Excel	Microsoft	RRID:SCR_016137
BioRender	Science Suite Inc.	RRID:SCR_018361; Graphical abstract created in BioRender: https://BioRender.com/r76w926

**Other**		

Digital table shaker	Southwest Science	SBT300
Forma series 3 water jacketed CO2 incubator	Thermo Scientific	4110
Stereomicroscope	Leica	M125 C and M60
Water bath	Fisher Scientific	FS-210
Precision balance	Fisher Scientific	01804318
Centrifuge	Beckman Coulter	B06320
Dumont#5 forceps	Fine Science Tools	91150-20
Spring scissors	Roboz Surgical	RS-5675
Vannas spring scissors	Fine Science Tools	15018-10

## Materials and equipment

**Table:** BSA 0.2% (20×)

Reagent	Final concentration	Amount
BSA	40 mg/mL	8 g
NaOH	N/A	∼1 mL
DPBS (with phenol red)	N/A	199 mL
Total	40 mg/mL	200 mL

Adjust final pH to ∼7.4. Sterile filter and store 1 mL aliquots at −20°C.

**Table:** BDNF (preparation of master stock: 1 mg/mL)

Reagent	Final concentration	Amount
BDNF	1 mg/mL	250 μg
0.2% BSA	N/A	250 μL
Total	1 mg/mL	250 μL

Store 80 μL aliquots at −80°C for up to 12 months for PeproTech products and up to 3 months for STEMCELL products.

**Table:** BDNF (preparation of working stock: 50 μg/mL)

Reagent	Final concentration	Amount
BDNF Master Stock	50 μg/mL	125 μL
0.2% BSA	N/A	2.375 mL
Total	50 μg/mL	2.3 mL

Store 80 μL aliquots at −80°C for up to 12 months for PeproTech products and up to 3 months for STEMCELL products.

***Alternatives:*** Alternatively, our research group has successfully used BDNF from STEMCELL technologies, 78133.

**Table:** NGF (nerve growth factor 2.5S) master stock

Reagent	Final concentration	Amount
NGF	1 mg/mL	1 mg
dd H_2_O	N/A	1 mL
Total	1 mg/mL	1 mL

**Table:** NGF (nerve growth factor 2.5S) 1000× aliquots

Reagent	Final concentration	Amount
NGF Master Stock	100 μg/mL	200 μL
0.2% BSA in DPBS	N/A	1800 μL
Total	100 μg/mL	2 mL

Store at −80°C for up to 3 months for Bio-Rad and STEMCELL products.

***Alternatives:*** Alternatively, our research group has successfully used NGF from STEMCELL technologies, 78151.

**Table:** NT-3 master stock

Reagent	Final concentration	Amount
Recombinant Human NT-3	0.1 mg/mL	50 μg
0.2% BSA in DPBS	N/A	450 μL
ddH_2_O	N/A	50 μL
Total	0.1 mg/mL	500 μL

**Table:** NT-3 working

Reagent	Final concentration	Amount
NT3 Master Stock	1 μg/mL	50 μL
0.2% BSA in DPBS	N/A	4950 μL
Total	1 μg/mL	5 mL

Store at −80°C for up to 3 months for PeproTech and STEMCELL products.

***Alternatives:*** Alternatively, our research group has successfully used NT3 from STEMCELL technologies, 78074.

**Table:** (12,500 Units/mL)

Reagent	Final concentration	Amount
Deoxyribonuclease I	12,500 units/mL	100 mg
EBSS	N/A	48 mL
Total	2.08 mg/mL	48 mL

Sterile filter with a 0.22 μm filter and store 200 μL aliquots at −20°C. Manufacturer recommends storing single use aliquots in −20°C or 70°C for up to one year.

**CRITICAL:** DNase I: May cause allergy or asthma symptoms or breathing difficulties if inhaled. May cause damage to organs in the respiratory tract if prolong and repeated exposure. Handle in a ventilated hood and wear respiratory protection if inadequate ventilation.

**Table:** Insulin

Reagent	Final concentration	Amount
Insulin	0.50 mg/mL	50 mg
1 N HCl	N/A	∼500 μL
ddH_2_O	N/A	99.5 mL
Total	0.50 mg/mL	100 mL

Sterile filter with a 0.22 μm filter and store 800 μL aliquots at −20°C.

**CRITICAL:** HCl: May cause severe skin burns and eye damage. May Cause respiratory irritation. Handle in a ventilated hood and wear protective gloves, clothing, eye protection, and face protection.

**Table:** Low-ovomucoid (low-ovo, 10×)

Reagent	Final concentration	Amount
DPBS	N/A	199 mL
Trypsin Inhibitor	15 μg/mL	3 mg
BSA	15 μg/mL	3 mg
NaOH	N/A	∼1 mL
Total	N/A	200 mL

Adjust final pH to ∼7.4. Sterile filter and store 1 mL aliquots at −20°C.

**CRITICAL:** Trypsin Inhibitor: May cause allergy or asthma symptoms or breathing difficulties if inhaled. May cause an allergic skin reaction. Handle in a ventilated hood and wear respiratory protection if inadequate ventilation. Wear protective clothes and gloves. NaOH: May cause severe skin burns and eye damage. Is harmful to aquatic life. Wear protective gloves/clothing/eye protection/face protection. Avoid release into the environment.

**Table:** High-ovomucoid (high-ovo, 6×)

Reagent	Final concentration	Amount
DPBS	N/A	198.5 mL
Trypsin Inhibitor	30 μg/mL	6 g
BSA	30 μg/mL	6 g
NaOH	N/A	∼1.5 mL
Total	N/A	200 mL

Adjust final pH to ∼7.4. Sterile filter and store 1 mL aliquots at −20°C.

**CRITICAL:** Trypsin Inhibitor: May cause allergy or asthma symptoms or breathing difficulties if inhaled. May cause an allergic skin reaction. Handle in a ventilated hood and wear respiratory protection if inadequate ventilation. Wear protective clothes and gloves. NaOH: May cause severe skin burns and eye damage. Is harmful to aquatic life. Wear protective gloves/clothing/eye protection/face protection. Avoid release into the environment.

**Table:** NAC (N-Acetyl-L-Cysteine)

Reagent	Final concentration	Amount
NAC	5 mg/mL	50 mg
Neurobasal	N/A	10 mL
Total	5 mg/mL	10 mL

Filter through pre-rinsed 0.22 μm filter and store 80 μL aliquots at −20°C.

**CRITICAL:** NAC: Causes serious eye irritation. Wear eye protection/face protection when handling.

**Table:** Borate buffer (pH 8.4)

Reagent	Final concentration	Amount
0.15 M Boric Acid	9.27 mg/mL	4.637 g
1 N NaOH	N/A	∼3 mL
ddH_2_O	N/A	497 mL
Total	9.27 mg/mL	500 mL

Store at 4°C.

**CRITICAL:** Boric Acid: May damage fertility or unborn child. Harmful to aquatic life. Wear protective gloves/clothing/eye protection/face protection. Avoid release into the environment. NaOH: May cause severe skin burns and eye damage. Harmful to aquatic life. Wear protective gloves/clothing/eye protection/face protection. Avoid release into the environment.

**Table:** PDL-Borate

Reagent	Final concentration	Amount
Poly-D-Lysine Hydrobromide (PDL)	1 mg/mL	50 mg
Borate Buffer	N/A	50 mL
Total	1 mg/mL	50 mL

DO NOT Filter. Store 200 μL aliquots at −20°C. Manufacturer guidelines suggest that the product is stable 2–3 years when stored at 2°C–8°C.

**CRITICAL:** PDL: To the best of our knowledge, no chemical, physical, or toxicological properties are known.

**Table:** Sato (100×)

Reagent	Final concentration	Amount
Progesterone Stock	60 ng/mL	25 μL
Sodium Selenite Stock	40 ng/mL	1000 μL
Neurobasal Medium	N/A	98.975 mL
Transferrin	100 μg/mL	1.0 g
BSA	100 μg/mL	1.0 g
Putrescine	16 μg/mL	160 mg
Total	N/A	100 mL

Store at −20°C.

**CRITICAL:** Progesterone: Suspected of causing cancer. May damage fertility or the unborn child. May cause harm to breast-fed children. Avoid contact during pregnancy/while nursing. Do not breathe dust or mist. Wear protective equipment while handling. Sodium Selenite: Fatal if swallowed or inhaled. Causes skin irritation. May cause an allergic skin reaction. Causes serious eye irritation. Toxic to aquatic life with long lasting effects. Do not breathe dust. Use in a well-ventilated area. Wear protective equipment. Wear respiratory equipment. Avoid release into the environment. Putrescine: Fatal if inhaled. Harmful if swallowed. Toxic in contact with skin. Causes severe skin burns and eye damage. Do not breathe dust/fume. Use in a well-ventilated area. Wear respiratory protection. Wear protective equipment while handling.

**Table:** T3 (3,3′,5-Triiodo-L-thyronine sodium salt 100×)

Reagent	Final concentration	Amount
T3 Solution (3.2 mg of powdered T3 in 400 μL of 1 N NaOH)	4 μg/mL	75 μL
DPBS	N/A	150 mL
Total	4 μg/mL	150 mL

Filter through pre-rinsed 0.22 μm filter and store 200 μL aliquots at −20°C.

**CRITICAL:** T3: Harmful if swallowed. Causes skin irritation. Causes serious eye damage. Very toxic to aquatic life with long lasting effects. Avoid release into environment. Wear protective equipment while handling.

**Table:** Tris pH 9.5 (1 M lab stock)

Reagent	Final concentration	Amount
Tris Base	1 M	60.57 g
ddH_2_O	N/A	<500 mL
10 N HCl	N/A	∼85 drops
Total	1 M	500 mL

Store at 4°C.

**Table:** Tris pH 9.5 (50 mM working stock)

Reagent	Final concentration	Amount
1 M Tris Stock	50 mM	50 mL
ddH_2_O	N/A	950 mL
Total	50 mM	1000 mL

Store at 4°C.

**CRITICAL:** Tris Base: To the best of our knowledge, no chemical, physical, or toxicological properties are known. HCl: May cause severe skin burns and eye damage. May Cause respiratory irritation. Handle in a ventilated hood and wear protective gloves, clothing, eye protection, and face protection.

**Table:** Pen-Strep (100×)

Reagent	Final concentration	Amount
Pen-Strep	N/A	100 mL
Total	N/A	100 mL

Store 800 μL aliquots at −20°C. Recommended shelf life of the product is 12 months from the date of manufacture.

**CRITICAL:** Pen-Strep: May cause an allergic skin reaction. Suspected of damaging fertility or the unborn child. Avoid breathing mist or vapor. Wear protective equipment while handling.

**Table:** Sodium Pyruvate

Reagent	Final concentration	Amount
Sodium Pyruvate	N/A	100 mL
Total	N/A	100 mL

Store 5 mL aliquots at 4°C. Recommended shelf life of the product is 12 months from the date of manufacture.

**CRITICAL:** Sodium Pyruvate: May cause an allergic skin reaction. Causes serious eye irritation. Avoid breathing dust. Wear protective equipment while handling.

**Table:** Glutamine (100×)

Reagent	Final concentration	Amount
Glutamine	N/A	100 mL
Total	N/A	100 mL

Store 800 μL aliquots at −20°C. Recommended shelf life of the product is 24 months from the date of manufacture.

**Table:** Cultrex mouse laminin I (500×)

Reagent	Final concentration	Amount
Cultrex Mouse Laminin I	N/A	1 mL
Total	N/A	1 mL

Store 10 μL aliquots at −80°C.

**Table:** N21-MAX (50×)

Reagent	Final concentration	Amount
N21-MAX	N/A	10 mL
Total	N/A	10 mL

Store 400 μL aliquots at −20°C.

**CRITICAL:** N21-MAX: Contains sodium selenite. Fatal if swallowed or inhaled. Causes skin irritation. May cause an allergic skin reaction. Causes serious eye irritation. Toxic to aquatic life with long lasting effects. Do not breathe dust. Use in a well-ventilated area. Wear protective equipment. Wear respiratory equipment. Avoid release into the environment.

## Step-by-step method details

### Dissection of DRGs

**Timing: 2–3 h**

This section outlines the process of extracting the embryos and isolating the DRGs from the spinal columns. Please see Methods video 1: Dissection of DRGs for the procedure.

1. Euthanize a female rat pregnant with E15 embryos. Our lab uses CO_2_ inhalation followed up with cervical location.
2. Extract embryonic sacs.
  a. Lay pregnant rat onto dissection area ventral side up.
  b. Spray ethanol on lower stomach.
  c. Using one hand, lift skin above stomach with Dumont #5 forceps (Fine Science Tools, 91150-20) and with the other, cut an incision along the midline with scissors (Roboz Surgical, RS-6702).
  d. Follow up with two incisions angled towards the hind legs to expose the embryonic sacs within the uterine wall.
  e. Carefully follow the uterine lining where the sacs connect and severe the connection.
  f. Cut away any fatty tissue and once the sacs have been isolated, gently lift and move all the embryos to the 15 cm Petri dish prepared with L15 media.
3. Remove embryos from embryonic sacs.
  ***Note:*** A litter typically consists of 10–15 embryos.
  a. Using forceps to pinch the embryonic sacs and with scissors (Roboz Surgical, RS-5675), cut the sac where the forceps are pinching.
    **CRITICAL:** Cut parallel to the tissue and avoid angling the scissors down and puncturing the embryo inside the sac.
  b. Repeat this until the embryo is exposed and using the forceps, gently push the sides of the embryonic sac so the embryo is squeezed out.
  c. With the scissors, cut the umbilical cord and disconnect the embryo from the sac.
  d. Use the forceps to scoop the embryo onto curved scissors and carefully transfer it to the 10 cm Petri dish prepared with L15 media.
  e. Repeat for every embryo.
4. Isolate spinal column with DRGs attached.
  ***Note:*** Please refer to Methods video S1 for a demonstration of the dissection steps. We perform these steps on one embryo at a time.
  a. Decapitate the embryo.
    i. Use forceps to hold the embryo in place.
    ii. Using scissors, cut at a 45° angle from the top of the spine down toward the chin.
  b. Once the embryo has been decapitated, cut away the tail and limbs.
  c. Put the embryo on its back, ventral side up, and cut off internal organs.
    ***Note:*** When cutting off the internal organs, make sure to cut parallel to the spine rather than down to avoid damaging the spinal cord. The ribcage and vertebrae should be visible at the end of this stage.
  d. Cut off the vertebrae from the ventral side with scissors.
    ***Note:*** This step helps with releasing DRGs from the spinal column at later stages. Completely reveal the spinal cord by removing the vertebrae that encapsulates the spinal cord.
  e. Flip the embryo dorsal side up.
  f. Find the rostral end and insert forceps in the space between the skin and the top of the spinal cord.
  g. In a zig-zag motion, use two forceps to incrementally peel the skin off along the length of the spinal cord.
  h. Place the forceps between the DRGs and the tissue on the lateral sides and use the other forceps to gently pull the tissue away, leaving just the spinal cord with DRGs attached for this step.
    **CRITICAL:** Be very gentle as the tissue at this stage is fragile. It is easy to accidentally rip off part of the spinal cord and lose the attached DRGs.
    ***Note:*** Once the skin has been peeled off, the DRGs should be visible on the side of the spinal cord.
  i. Transfer the spinal cord to the 5 cm Petri dish with DPBS and repeat steps for every embryo.
5. Collect DRGs.

***Note:*** Please refer to Methods video 1 for a demonstration of this step.

6. Focusing on one at a time, use forceps to brace the spinal cord and another pair of forceps to pinch at the base of the DRGs.

***Note:*** Another way to remove DRGs is to use Vannas Spring Scissors (Fine Science Tools, 15018-10) to cut away the DRGs while still anchoring the spinal cord with a pair of forceps.

7. Work along entire column to ensure there are no remaining DRGs attached.

***Note:*** Be careful to just collect DRGs and no other tissues.

***Note:*** Cooling neurons to temperatures lower than 18°C–25°C room temperature is not recommended, as it can decrease cell viability.^9^

8. Discard the stripped spinal cord and once the DRGs have been collected from all the embryos, go back and remove tissues other than DRGs. Also see troubleshooting.
9. In the fume hood, transfer DRGs into a 15 mL Falcon tube by decanting.
10. Wait 5 min for DRGs to settle to the bottom, then use a serological pipette to remove L-15 media from the top.

### Dissociation

**Timing: 1 h**

These steps describe dissociation of DRGs using a papain solution and trituration of cells in the ganglia for obtaining a single cell suspension prior to panning steps.

11. Add 4 mL of DPBS to DRGs to wash out L15 media.
12. While DRGs settle, prepare the papain solution.
13. Use the tube previously placed in the 37°C water bath (Preparation on the day of the DRG Immunopanning, step 17) and add 100 units of papain to the solution.
14. Once papain has been added, return it to the water bath for a few min to allow the papain to dissolve.
15. Retrieve L-cysteine and measure out 0.004 g.
16. Remove papain tube from water bath, add L-cysteine to solution, and bring into biosafety cabinet.
17. Filter solution through 0.22 μm syringe filter into new, labeled 15 mL Falcon tube.
18. Add 100 μL of DNase, close lid, and invert tube to mix solution thoroughly.

***Note:*** Save remaining DNase to make Lo-ovomucoid (low-ovo) solution at later step.

19. Remove DPBS from tube containing DRGs using a serological pipette and add the entire papain solution.
20. Place tube with DRGs and papain solution into water bath to incubate for 30 min.

**CRITICAL:** Take the tube out at the halfway point to mix the solution inside by gently flicking 2–3 times, then return it to the bath.

21. While the DRG/papain mixture incubates in the water bath, prepare following solutions.
22. To prepare the low-ovo, combine 9 mL DPBS without calcium/magnesium + 1 mL low-ovo stock solution + 100 μL of DNase.

***Note:*** Add DNase right before use.

23. To prepare the high-ovomucoid (high-ovo) solution, combine 5 mL DPBS + 1 mL high-ovo stock solution + 3 μL 1 M NaOH.
24. To prepare the panning buffer, combine 18 mL DPBS + 2 mL 0.2% BSA + 200 μL insulin.
25. Remove DRG/papain solution from water bath after 30 min and bring back into biosafety cabinet.
26. Carefully wash DRGs.
27. Let the DRGs settle down to the bottom of the falcon tube. Remove the papain solution using a serological pipette, without aspirating the DRGs.
28. Add 4 mL low-ovo solution.

***Note:*** It is recommended to place the pipette tip against the inner wall of the Falcon tube and release the solution slowly to be as gentle as possible.

29. Wait 1 min for DRGs to settle to the bottom of the tube, then remove the 4 mL low-ovo solution.
30. Triturate DRGs.
  a. Add 1 mL low-ovo solution to DRGs with a P1000 pipette.
  b. Keep pipette tip inserted into solution and very slowly pipette up and down 8-10 times, which will make the low-ovo solution cloudy as the cells go into suspension.
    **CRITICAL:** Be careful to avoid bubbles by keeping the pipette tip inserted into liquid.
  c. Wait 1-2 min for large tissue chunks to settle to the bottom.
  d. Transfer cloudy top part of the low-ovo solution to new 50 mL Falcon tube without disturbing the settled down DRG tissue.
  e. Add another 1 mL low-ovo solution and repeat steps (a-d) until all DRGs are dissociated.
    ***Note:*** Optional to count cells by trypsin blue exclusion during centrifugation step by diluting the cells 1:2 with Trypsin Blue (Thermo Fisher, 15250061)
31. Use a 10 mL serological pipet (Fisher Scientific, 13-675-20) to slowly add 6 mL high-ovo solution under low-ovo solution, leading to a clear layer at the bottom and a cloudy cell suspension on top.
32. Pellet the cells for 10 min at 220 G at 18°C–25°C.
33. While cells are in the centrifuge, make a cone to filter cells.
  a. Use forceps to place a 20-micron nylon mesh filter (Sefar, 03–20/14) onto 50 mL Falcon tube.
  b. With the tip of 1 mL pipette, press into the filter to make cone shape and wet filter with 1 mL panning buffer.

***Note:*** Please see Methods video S2: Making a cone for cell filtering for this step.

34. Once centrifugation has finished, discard the low-ovo and high-ovo solutions on the cell pellet via suctioning with a glass Pasteur pipette.

**CRITICAL:** Be very careful not to disturb the DRG cell pellet by avoiding inserting the Pasteur pipette beyond the 5 mL marking on the Falcon tube. Instead, gently decant the solution to feed it into the suction. The cell pellet will not be visible.

35. Resuspend the DRG cells pellet.
  a. Add 1 mL panning buffer to DRG tube.
  b. Pipette up and down gently to mix and once cells are fully into the solution, add another 6 mL panning buffer.
36. Filter solution through Nitex cone 1 mL at a time.
37. Wash Nitex cone with 2 mL panning buffer.
38. Place filtered solution into 37°C, 10% CO2 incubator for 30 min.

**CRITICAL:** Keep screw cap loose on Falcon tube to allow gas exchange.

### Immunopanning and plating cells

**Timing: 2 h**

**Figure 1:**
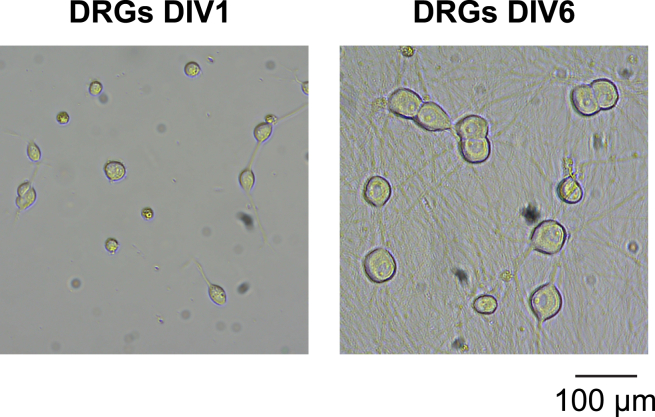
Highly pure DRG neuron cultures obtained via immunopanning Bright field images (taken with an ACCU-SCOPE EXI-310-PH Inverted Microscope using a 20× objective) show DRG neurons at 1 and 6 days in vitro (DIV1 and DIV6) after immunopanning. Scale bar represents 100 μm.

This step involves using two negative panning plates to purify the DRG neurons. First, the BSL plate will remove blood and endothelial cells, then the CD9 plate will remove remaining glial cell types. These immunopanning steps remove most other cell types from the single cell suspension and yields a more then 97% pure DRG neuronal culture (Figure 1).

39. Rinse BSL-1 panning plate with ∼10 mL DPBS 3 times then pour out remaining solution from plate. Do not allow the panning plates dry out in between the washing steps.
40. Pour filtered cell suspension onto BSL-1 plate.
41. Keep the BSL-1 plate outside of the cell culture hood, on a flat, non-vibrating surface, at room temperature for 20 min, and gently shake the plate halfway through.

***Note:*** Plate must be shaken front-back and side-to-side, not in a swirling motion, otherwise, all cells will accumulate at the center instead of spreading across the surface of the dish.

42. Rinse CD9 panning plate with ∼10 mL DPBS 9 times then pour out remaining solution from plate.

**CRITICAL:** It is important to wash the plate this many times because the antibody contains sodium azide, highly toxic to cells.

43. Transfer the unbound cells from BSL-1 to the CD9 panning plate.
44. Shake BSL-1 plate forward and backward, side-to-side then pour solution into CD9 plate.
45. Tilt BSL-1 plate down and pipette remaining solution at an angle using a 1 mL pipette to transfer to CD9 plate.
46. Keep the CD9 plate outside of the cell culture hood, on a flat, non-vibrating surface, at room temperature for 30 min, and gently shake the plate halfway through.

***Note:*** Plate must be shaken front-back and side-to-side, not in a swirling motion, otherwise, all cells will accumulate at the center instead of spreading across the surface of the dish.

47. Gently shake the CD9 plate and transfer DRG cell suspension into 50 mL Falcon tube.

***Note:*** Just like in step 25, tilt the plate and use a 1 mL pipette to transfer remaining solution into tube.

48. Take the DRG media out of the 4°C fridge and place into 37°C incubator for 15 min to warm until addition of growth factors.
49. Count cells (refer to Dissociation, step 30).
  a. Dilute cell suspension with Trypsin Blue 1:2.
  b. Use a hemacytometer to count the number of cells.

***Note:*** For 10–15 embryos, expect around 1 × 10^6^ to 2 × 10^6^ cells.

50. Centrifuge for 10 min at 220 G at 18°C–25°C.
51. In preparation for plating, take coverslips with laminin out from incubator and use Dumont N7 curved tweezers (Fisher Scientific, 5024289) to transfer them to 24-well plates.
52. Add growth factors to warm DRG medium as follows:
  a. 1:1000 dilution BDNF stock (BDNF) (STEMCELL Technologies, 78133).
  b. 1:1000 dilution NT3 stock (NT3) (STEMCELL Technologies, 78074).
  c. 1:1000 dilution NGF-beta stock (NGF) (STEMCELL Technologies, 78151).

***Note:*** Growth factors are stored in the −80°C.

53. After centrifugation, resuspend DRG cell pellet at 1000 cells per μL.
54. Aspirate the remaining laminin solution from the coverslips transferred to 24-well plates in before you begin Step 6.
55. Pipet 5-10 μL of the cell suspension onto the center of each coverslip and incubate for 5-10 min at 18°C–25°C to allow the cells to attach the coverslips.
56. Flood each well with 500 μL DRG media with growth factors.

***Note:*** Do this in a circular motion around the edge of the coverslips to avoid disturbing the DRGs.

57. Place lid onto 24 well plate and keep in 37°C incubator up to 2–3 weeks.

***Note:*** Media tends to evaporate at a higher rate on the edges of the well plate, so to offset that put a small amount of autoclaved PBS in between wells.

### Feeding DRG neurons

**Timing: 30 min**

Cells require fresh media change every 2–3 days for optimal cell survival and health (Figure 1). Prepare fresh DRG base with growth factors in a Falcon.

**CRITICAL:** For the first feed only, the next day of the prep, prepare 5-fluoro-2-deoxyuridine (FUDR) at 20 μM in DRG medium + growth factors. This will kill any remaining contaminating cells.

58. Warm the DRG media in the incubator for 5–10 min with a loose cap to allow gas exchange.
59. Take the well plate and media out of incubator and do half-media change.

***Note:*** Remove 200 μL of media from wells and replace with 250 μL freshly prepared media.

60. Return plate to incubator.
61. Feed cells every 2–3 days.

### Immunofluorescence for cultured DRG neurons

**Timing: 2 days**

The following protocol can be used for immunofluorescence of DRG neurons, detection of a protein of interest, and measurement of fluorescence intensity using Cellpose.^8^

62. Fix cells.
  a. Prepare Fixing Solution: Prepare 4% Paraformaldehyde (PFA) (Electron Microscopy Sciences, 15711) solution in PBS.
  b. Carefully remove media from each well using P1000 pipettes.
  c. Add 500 μL of 4% PFA solution into each well and let sit for 20 min.
  d. Remove fixation solution and rinse with PBS 3 times.
63. Prepare Blocking Solution: 1% Triton (Sigma-Aldrich, 93443), 5% Normal Goat Serum (Fisher Scientific, PCN5000) and 5% Normal Donkey Serum (Fisher Scientific, NC2039255) containing solution in PBS.
64. Add 500 μL blocking solution to each well of 24 well-plate and leave at room temperature for 1 h.
65. Prepare Antibody Solution:
  a. Prepare a 0.1% Triton (Sigma-Aldrich, 93443), 0.5% Normal Goat Serum (Fisher Scientific, PCN5000) and 0.5% Normal Donkey Serum (Fisher Scientific, NC2039255) containing solution in PBS for primary and secondary antibody incubation.
  b. Add the primary antibody at desired dilution factor.
  c. Close lid tightly and invert the tube a few times to mix antibody throughout solution.

***Note:*** Antibodies tested with this protocol^1^ and their dilution factors are indicated in the key resources table.

66. Remove blocking solution and add 200 μL primary antibody solution to each well.
67. Fill holes between wells with small amount of PBS to reduce evaporation, and place lid on plate.
68. Parafilm the 24 well-plate and incubate at 4°C ≥16 h.
69. The following day, prepare PBS with 0.1% Triton (PBST).
70. Remove primary antibody solution from wells and discard.
71. Wash with prepared PBST 3 times with 20-min intervals.

***Note:*** Set well-plate on table shaker and turn on lowest setting during 20-min intervals.

72. During last interval, prepare secondary antibody solution by adding the appropriate Alexa Fluor secondary antibody with a 1:1000 dilution to antibody solution from Step 65.

**CRITICAL:** Prepare solution right before use because it is photo-sensitive.

73. Remove the PBST from wells.
74. Add 250 μL secondary antibody solution to each well and let it sit at 18°C–25°C for 1 h on the bench.

**CRITICAL:** Cover well-plate with aluminum foil to protect antibodies from light.

75. Repeat step 71.

**CRITICAL:** Make sure plate is covered with foil during 20-min intervals.

76. Put PBS in wells.
77. Mount coverslips onto slides for imaging.
78. Onto each Super frost slide (Fisher Scientific, 12550143), place one droplet of Prolong Gold antifade reagent with DAPI (Fisher Scientific, P36930) per coverslip.
79. Using Dumont N7 tweezers (Fisher Scientific, 5024289), transfer cover slips onto slides cell side down.
80. Use Kimwipe to remove excess liquid around edges.
81. Apply clear nail polish (for example, Sally Hansen topcoat nail hardener [Amazon, B07JB2C8YN]) around edges of coverslips, careful to avoid getting polish close to center of cover slip.
82. Place slides flat inside a slide box and allow the coverslips to dry for at least 30 min at 18°C–25°C.
83. Transfer slide box to 4°C fridge.
84. Our research group image cells using a Zeiss LSM 800 laser scanning confocal microscope with Zeiss Zen Blue software. We save images as CZI files and convert to TIFF format before analysis.^1^
85. Run Cellpose analysis.^8^
86. After downloading Cellpose^8^ based on the instructions, open the Terminal and enter the following commands:

Conda

conda activate/opt/anaconda3/envs/cellpose

cellpose

87. Click and drag a file onto Cellpose image portal.
88. Choose the channel to segment and run the analysis.
89. Modify parameters to obtain region of interests that detect the cell body of DRG neurons.

**CRITICAL:** Press command + n to save that image as “cp_masks.png”. This saves the detected cell masks.

90. Measure the fluorescent intensity in the ROIs detected by Cellpose using the “Label to ROIs” function in Fiji.
91. Use a batch renaming software to rename all the files automatically and change the automatically saved mask names from “_cpmasks.png” to “_label.png” as “label” must be included in the mask name for the “Label to ROIs” Fiji plug-in to work.
92. Open Image J and run “Label to ROIs”
93. Select “multiple images” and find the folder including the raw image and image mask files and run the analysis. “Full_results_table_Erosions” file will contain results from all the images within the folder.

### ACD RNAscope procedure: Multiplex fluorescent v.2 assay

**Timing: 3 days**

The following protocol describes in situ hybridization of cultured DRG neurons for mRNA detection using RNAscope and quantification of RNAscope results using Fishquant.^7^

94. Fix Cells.
  a. Prepare 4% paraformaldehyde (PFA) (Electron Microscopy Sciences, 15711) solution in PBS.
  b. Carefully remove media from each well using P1000 pipettes.
  c. Add 500 μL of 4% PFA solution into each well and let sit for 20 min.
  d. Remove fixation solution and rinse with PBS 3 times.
  e. Remove the PBS and add 250 μL of 50% ethanol for 1 min.
  f. Repeat step (e) with 70% then 100% ethanol.
  g. Replace the 100% ethanol with 1 mL of fresh 100% ethanol.

***Note:*** These cover glasses can be stored in 100% ethanol for up to 6 months at −20°C. Please see the note below before starting the RNAscope procedure.

95. RNAscope multiplex fluorescence kit (ACDbio, 320851) were used to detect target mRNA targets (such as Rn-Scn8a as the C1 Probe, Scn10a as the C2 probe, and Scn9a as the C3 probe) by following manufacturer’s protocol in this link.

**Figure 2:**
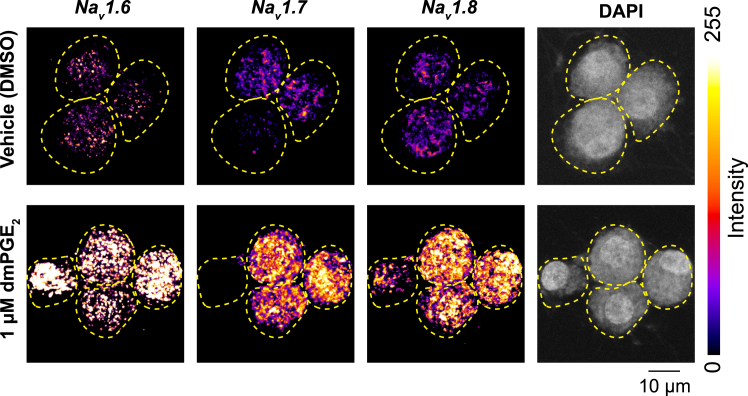
RNAscope labels target transcripts in immunopanned DRG neurons RNAscope shows the expression of *Na*_*v*_*1.6*, *Na*_*v*_*1.7* and *Na*_*v*_*1.8* in DRG neurons isolated using the protocol described here, cultured for 9 days in vitro (DIV), and either treated with DMSO or 1 μM 16,16-dimethyl PGE_2_ (dmPGE_2_)—a PGE_2_ derivative that is resistant to degradation on DIV8 for 16-24 h. Scale bar represents 10 μm.

***Note:*** This kit has recently been discontinued and replaced with ACDbio, 323100. We successfully implemented this new kit for mRNA detection in DRG neurons (Figure 2).

***Note:*** It is essential to dry cells on coverslips for 20 min at 18°C–25°C to ensure cells remain attached to coverslips during the RNAscope protocol.

***Note:*** For the RNAscope Protease III step, use Protease IV instead and leave it in the Humidity Control Tray at 18°C–25°C for 30 min. In our pilot tests, Protease III did not work in RNAscope assays for cultured DRG neurons. Protease IV incubation for 30 min provides robust, reproducible RNAscope staining of target mRNAs.

***Note:*** Our research team perform confocal imaging using a Zeiss LSM 800 laser scanning confocal microscope with Zeiss Zen Blue software (Figure 2). We use the Tiles function to set up multiple positions to image many DRG neurons in one imaging experiment. To capture all mRNAs detected, we use the Z-stack function.

96. *FISHQuant* Analysis Set-Up.
  a. Install *MATLAB, Fiji,* and *Name Mangler* onto a computer.
  b. Search for the *FISHQuant MATLAB* Code (https://bitbucket.org/muellerflorian/fish_quant/src/master/).
  c. Install the package.
  d. Install the macro provided below into Fiji.

input_path2=getDirectory("Choose C1 folder");

input_path3=getDirectory("Choose C2 folder");

input_path4=getDirectory("Choose C3 folder");

input_path5=getDirectory("Choose C4 folder");

input_path1 = getDirectory("Choose image folder");

fileList = getFileList(input_path1){;

for (f=0; f<fileList.length; f++){

open(input_path1 + fileList[f]);

//splits channels and renames channel titles

title = getTitle ;

run("Split Channels");

selectWindow("C1-"+title);

rename("C1-"+title);

selectWindow("C2-"+title);

rename("C2-"+title);

selectWindow("C3-"+title);

rename("C3-"+title);

selectWindow("C4-"+title);

rename("C4-"+title);

//count particles for Channel 1

selectWindow("C1-"+title);

saveAs ( "tiff" , input_path2+"c1-"+title ) ;

//count particles for Channel 2

selectWindow("C2-"+title);

     saveAs ( "tiff" , input_path3+"c2-"+title ) ;

//count particles for Channel 1

selectWindow("C3-"+title);

     saveAs ( "tiff" , input_path4+"c3-"+title ) ;

//count particles for Channel 2

selectWindow("C4-"+title);

     saveAs ( "tiff" , input_path5+"c4-"+title ) ;

      run("Close All");

}

***Note:*** This macro divides up CZI Z-Stack photos into their respective channels. If you prefer to use a different program to accomplish that goal, that should suffice.

97. FISHQuant analyzes RNAscope images one channel at a time and only works with images with a single channel. Thus, we do the following steps to split channels after our imaging session on Zen software.
  a. Create a new folder marked uniquely to describe your experiment,
  b. Within that folder, create four new folders named "C1", "C2", "C3", "C4”.
  c. Open Fiji. Open the macro above in the Fiji software.
  d. Go to File and find “Bio-Formats (windowless)”.
  e. Select one of your photos and click “ok.” While running the macro, Bio-Formats will use the same settings while importing images.
  f. Click "Run" and the program will ask you to select four folders to save the images of individual channels. Select C1 folder, C2, C3, the C4 folder, and finally the folder where your images are.
  g. The program should have split each one of your channels into the appropriate folder.
98. Create cell outlines using FISHQuant.
  a. Open *MATLAB* and type “FQ” into the main data frame.
  b. Go to the Folders tab. Click “Folder for Images” and select the C1 Folder. Then click “Folder for Outlines” and create a new folder labeled “Outlines”.
  c. Click Tools and click “List Directory Content”.
  d. Click on the first image. This will bring up a new tab of the image for that specific channel.
  e. Click “New Cell” and trace around a full cell. Then continue until all the cells are traced.
    ***Note:*** To add the DAPI channel to the background, copy and paste all the DAPI photos into the image folder you are accessing (For example, C1, C2, or C3). Then click “Load Images”, “Load DAPI”. A pop-up will be there, and you put the channel you are using initially in the first row and “DAPI” into the DAPI row.
  f. After circling all the cells, save that file to the outline folder. Repeat these steps until you have created an outline for every image.
99. Optimize FISHQuant analysis settings for each channel.
  a. Go back to the main interface page of FISHQuant and click “load image“.
  b. Select one of the images from the channel folder you are using.
  c. Click “Define Outlines”.
  d. Trace around a cell but make it large enough to contain some areas without staining.
    ***Note:*** This outline will help with correctly selecting the threshold for quantifying RNAscope signal.
  e. Click “Finished”.
  f. Click “Detect” and click “ok” to the first pop-up.
  g. Make sure “allow smaller region in Z for analysis” is clicked, then click “Perform detection”.
  h. Adjust the Threshold Slider until the green crosses are over all the mRNA dots but none that are background noises.
  i. Click “Apply detection”.
  j. Click “Fit” and “Apply Threshold”.
  k. Save the detection settings in a new folder named “Detection Setting” and make sure file name is unique to that channel.
100. Analyze image files using FISHQuant batch processing.
  a. Click “Tools” and click “Batch Processing”.
  b. Load the detection settings you saved and add all the cell outlines you created.
  c. Click “Process”.
  d. Save both the “Summary: Mature mRNA” and the “Analysis Results” to a new results folder.
101. Using Name Mangler or another batch renaming software, copy and paste the names of your C2 images.
102. Replace “C2” with “C1” and repeat these steps (99 and 100) but changing the root image folder to where the newly named C2 images are.

***Note:*** This will enable you to analyze other channel images using the same outlines, without the need to make outlines of the same cells for each channel.

## Expected outcomes

DRG neurons form the basis of somatosensation. They extend long projections into the skin, muscles, bones, and internal organs to relay sensory information, including touch, pain, cold, and itch to the central nervous system. In this protocol, we describe an efficient method for isolating DRG neurons in cultures that are more than 97% pure, largely devoid of glial cells or other cell types. Using a defined medium containing NGF, NT3, and BDNF growth factors, DRG neurons are grown under serum-free conditions.

Experimenters can expect to isolate approximately one million DRG neurons per preparation, using ten to fifteen embryos. Isolated neurons can be cultured for up to three weeks and used in pharmacological treatments, viral infections, live imaging experiments, or electrophysiology.^1^ In our previous studies, we have also isolated RNA from DRG neurons for a bulk RNA sequencing experiment to examine how DRG neurons respond to PGE_2_ and treatment with Schwann cell–secreted factors.^1^

Immunopanned neurons can be used for immunofluorescence or in situ hybridization assays (RNAscope), as described, to analyze protein or mRNA expression of target genes (Figure 2). We also describe methods for reliable, rigorous, and automated analysis of these experiments using Cellpose and FishQuant.^7^^,^^8^

In summary, this protocol enables the isolation of DRG neurons in highly pure cultures. The isolated cells can be used in various assays to investigate the molecular and physiological properties of DRG neurons.

## Limitations

This protocol enables generation of highly pure DRG neuron cultures from E15 rat embryos, largely devoid of other cell types.^1^ Isolated DRG neurons can be used to investigate neural mechanisms of sensory development, pain, and neurodevelopment disorders. However, the protocol is limited in its ability to obtain fully mature DRG neurons suitable for modeling mature sensory DRG neurons in vivo.

We previously conducted an RNA-seq experiment on DRG neurons isolated using the protocol described here.^1^ We found that these DRG neurons predominantly expressed progenitor and unspecialized sensory neuron markers and did not express markers specific to distinct DRG subtypes.^1^ This result suggests that DRG neurons fail to fully differentiate into mature subtypes in culture. Our future work will focus on determining whether neural activity and interactions with Schwann cells can induce subtype differentiation of DRG neurons in culture.

Second, our protocol is inefficient for isolating DRG neurons from rodents at postnatal stages. However, we have previously modified the digestion steps of this protocol by incorporating collagenase and dispase enzymes to aid in tissue dissociation, successfully isolating DRG neurons from postnatal (P18/P19) mice,^1^ similar to other studies that successfully isolated DRG neurons from postnatal, adult or aged mice.^10^^,^^11^^,^^12^ Similar modifications might allow adapting this protocol for isolating and culturing pure DRG neurons from adult rodents in future studies.

## Troubleshooting

### Problem 1

Contamination from other cell types in DRG neuron cultures (related to Step: Dissection of DRGs).

To ensure that DRG neuron cultures are pure, consider following the suggestions below.

### Potential solutions

• During last step of dissection when separating DRG neurons from spinal column, remove any small tissue pieces that are not DRGs using forceps.
• Ensure that CD9 panning plate is incubated at 18°C–25°C with CD9 antibody for at least 2 h.
• Do not skip the Dissociation step 38, to allow recovery of cells from dissociation and maximum presentation of antibody binding sites.

### Problem 2

Low cell viability and yield after the immunopanning steps.

DRG neurons may be over-digested with papain, resulting in reduction of cell viability (related to- Dissociation, step 13).

### Potential solutions

• Papain concentration: Try reducing the papain amount to 75 or 50 units in Dissociation step 13.
• If the dissociation steps are done too vigorously, it will result in destruction of cells. Be as gentle as possible during the trituration of the cells (Dissociation step 13) and pipette gently, slowly and without introducing bubbles.
• Include at least 10 embryos in the dissection steps, as cell viability decreases with less starting tissue material.

## Resource availability

### Lead contact

Further information and requests for resources and reagents should be directed to and will be fulfilled by the lead contact, Husniye Kantarci, husniye.kantarci@austin.utexas.edu.

### Technical contact

Technical questions on executing this protocol should be directed to and will be answered by the technical contact, Samuel Faraguna, samuel.faraguna@austin.utexas.edu.

### Materials availability

This study did not generate new unique reagents.

### Data and code availability

Original data for Figure 2 is deposited at Mendeley Data: https://data.mendeley.com/datasets/rxvfxkr6v9/1 Fiji macros used in the data analysis are included under the analysis steps. No other datasets or code is generated in this study.

## Acknowledgments

We thank Dr. J. Bradley Zuchero for support, encouragement, critical reading of the manuscript, and feedback. The graphical abstract was created in BioRender (Kantarci, H. [2025]; https://BioRender.com/r76w926). We thank the University of Texas at Austin and Brain & Behavior Research Foundation for their financial support.

## Author contributions

S.F. and K.A.P.: conceptualization, imaging and video capture, analysis, and writing. H.K.: methodology, supervision, writing, review, and editing.

## Declaration of interests

The authors declare no competing interests.

## Supplemental information

Methods video S1. Dissection of DRGs Video shows steps for dissection of DRGs from E15 rat embryos related to step: Dissection of DRGs.

Methods video S2. Making a cell–filtering cone Video shows steps for making a nylon mesh filter cone to filter dissociated cells prior to immunopanning, related to: Dissociation, step 36.
